# Angiotensin II induces endoplasmic reticulum stress in podocyte, which would be further augmented by PI3-kinase inhibition

**DOI:** 10.1186/s40885-015-0018-5

**Published:** 2015-07-08

**Authors:** Tae-Sun Ha, Hye-Young Park, Su-Bin Seong, Hee Yul Ahn

**Affiliations:** Department of Pediatrics, College of Medicine, Chungbuk National University, 1 Chungdae-ro, Seowon-gu, Cheongju, 361-240 South Korea; Department of Pharmacology, College of Medicine, Chungbuk National University, 1 Chungdae-ro, Seowon-gu, Cheongju, 361-240 South Korea

**Keywords:** Angiotensin II, Endoplasmic reticulum stress, Phosphoinositide 3-kinase, Podocyte

## Abstract

**Introduction:**

Angiotensin II (Ang II) contributes to the pathological process of vascular structures, including renal glomeruli by hemodynamic and nonhemodynamic direct effects. On renal effects, Ang II plays an important role in the development of proteinuria and glomerulosclerosis by the modification of podocyte molecules and cell survival. In the present study, we investigated the effect of Ang II on endoplasmic reticulum (ER) stress in podocytes.

**Methods:**

We cultured mouse podocytes with increasing doses of Ang II and evaluated ER stress markers by Western blotting.

**Results:**

Ang II increased Bip protein, an ER chaperone, in a dose-dependent manner at 24 h, which was ameliorated by losartan, an angiotensin II type 1 receptor antagonist. Ang II also increased ER stress markers, such as phospho-PERK, phospho-eIF2α, and ATF4 proteins of podocyte, significantly in a dose-dependent manner at 24 h. Increased phospho-PERK and ATF4 proteins were further augmented by phosphoinositide 3 (PI3)-kinase inhibitor, LY294002, which suggested that Ang II could induce podocyte ER stress of PERK-eIF2α-ATF4 axis via PI3-kinase pathway.

**Discussion:**

These studies suggest that Ang II could induce podocyte ER stress of PERK-eIF2α-ATF4 axis via PI3-kinase pathway, which would contribute to the development of podocyte injury induced by Ang II, and the augmentation of PI3-kinase would be a therapeutic target.

## Introduction

Angiotensin II (Ang II) directly constricts vascular smooth muscle cells, stimulates aldosterone production, activates the sympathetic nervous system, and increases sodium reabsorption, all of which are mediated through Ang II type 1 receptor (AT1R) activation and contributes to the development of hypertension [1,2]. In addition to its hypertensinogenic effect, locally produced Ang II in the kidney activates multiple signaling pathways and mediates inflammation, renal cell growth, mitogenesis, apoptosis, extracellular matrix accumulation, proteinuria, and glomerulosclerosis via the angiotensin II type 1 receptor (AT1R) [3-6]. These effects of Ang II play an important role in the pathogenesis of renal tissue injury.

The podocyte is a highly differentiated epithelial cell that situated on the outer surface of the glomerular basement membrane (GBM), playing a crucial role in the regulation of glomerular filtration function, and podocyte injury is an essential feature of proteinuria and progressive glomerular diseases [7-9]. The foot processes of neighboring podocytes regularly interdigitate, leaving between them the slit diaphragm (SD), which form the final layer of the glomerular filtration barrier and is linked to the actin-based cytoskeleton by adaptor proteins, including CD2-associated protein (CD2AP), zonula occludens (ZO)-1, β-catenin, etc. [7-9].

The endoplasmic reticulum (ER) is a highly dynamic organelle in which newly synthesized secretory and transmembrane proteins are assembled and folded into their correct tertiary structures, thereby, playing a critical role in controlling the fate of cells. The ER is a highly dynamic organelle responsible for multiple cellular functions. However, the ER is highly sensitive to alterations in its homeostasis. A number of conditions can disturb ER functions, and these conditions induce a state known as ER stress (ERS) [10,11].

ERS is defined as accumulation of unfolded or misfolded proteins in the ER, which triggers an adaptive program called the unfolded protein response (UPR) [12-14]. The adaptive UPR pathway is regulated by three major ER-resident transducers (ERS sensors), those are RNA-dependent pancreatic eukaryotic translation initiation factor 2α (eIF2α) kinase (PERK, PKR-like ER kinase), activating transcription factor 6 (ATF6), and inositol-requiring ER-to-nucleus signal kinase 1 (IRE1). Activation of PERK leads to phosphorylation of eukaryotic translation initiation factor 2α (eIF2α), which causes general inhibition of protein synthesis and induction of a transcription factor ATF4 that binds to the amino acid response element [12,13]. However, the ERS mechanisms underlying the development of podocyte injury by Ang II remain to be clarified. We hypothesized that PERK-eIF2α-ATF4 axis ERS would be involved in Ang II-induced podocyte injury.

## Methods

### Cell culture of mouse podocytes

Conditionally immortalized mouse podocytes were kindly provided by Peter Mundel (University of Harvard, Boston, MA, USA) and were cultured and differentiated as described previously [15]. Briefly, to stimulate podocyte proliferation, cells were cultivated at 33°C (permissive conditions) in a culture medium supplemented with 10 U/mL mouse recombinant γ-interferon (Roche, Mannheim, Germany) to induce expression of temperature-sensitive large T antigen. To induce differentiation, podocytes were maintained at 37°C without γ-interferon (non-permissive conditions) for at least 2 weeks.

### Treatment conditions and preparation of antibodies

Mouse podocytes were incubated with various concentrations of Ang II (Sigma-Aldrich Inc., St. Louis, MO, USA) for 24 h. To inhibit phosphoinositide 3(PI3)-kinase/Akt signaling, cells were exposed to 5 μM LY294002 (Cell Signaling Technology, Danvers, MA, USA), a PI3-kinase inhibitor. For Ang II inhibition, losartan (Merck, MSD LTD., Seoul, Korea) was used in a concentration of 10^−6^ M for 24 h. The primary antibodies for Western blotting were purchased as follows: anti-phospho-PERK and anti-β-tubulin antibodies from Santa Cruz Biotechnology (CA, USA) and anti-Bip, anti-ATF4, anti-phospho-eIF2α, and anti-eIF2α antibodies from Cell Signaling (Beverly, MA, USA).

### Western blotting

Total proteins from podocytes were extracted using protein extraction solution (PRO-PREP, Intron, Seongnam, Kyungki, Korea) according to the manufacturer’s instructions. Thirty micrograms of boiled extracts was applied on 10% or 12% SDS-PAGE gels and transferred to polyvinylidene fluoride membranes (Bio-Rad Laboratories, Hercules, CA, USA). Then, the membranes were air-dried and blocked in 3% fat-free milk before incubation with the primary antibodies. After incubation with horseradish peroxidase-conjugated secondary antibodies (Santa Cruz Biotechnology), bands were detected by using the enhanced chemiluminescence system (Amersham Biotech Ltd., Bucks, UK). In each experiment, the ratio of absorbance of each molecule vs. β-tubulin was calculated. Density values were expressed as percent of control (without Ang II).

### Statistical analysis

The results are expressed as mean values ± standard deviation. The statistical significance was assessed by nonparametric Kruskal-Wallis ANOVA analysis or Student’s *t*-test using the SPSS 9.0.0 (SPSS, Chicago, IL, USA) software program. *P* values less than 0.05 were considered significant.

## Results

### Ang II induces ER stress in podocyte

**Figure 1:**
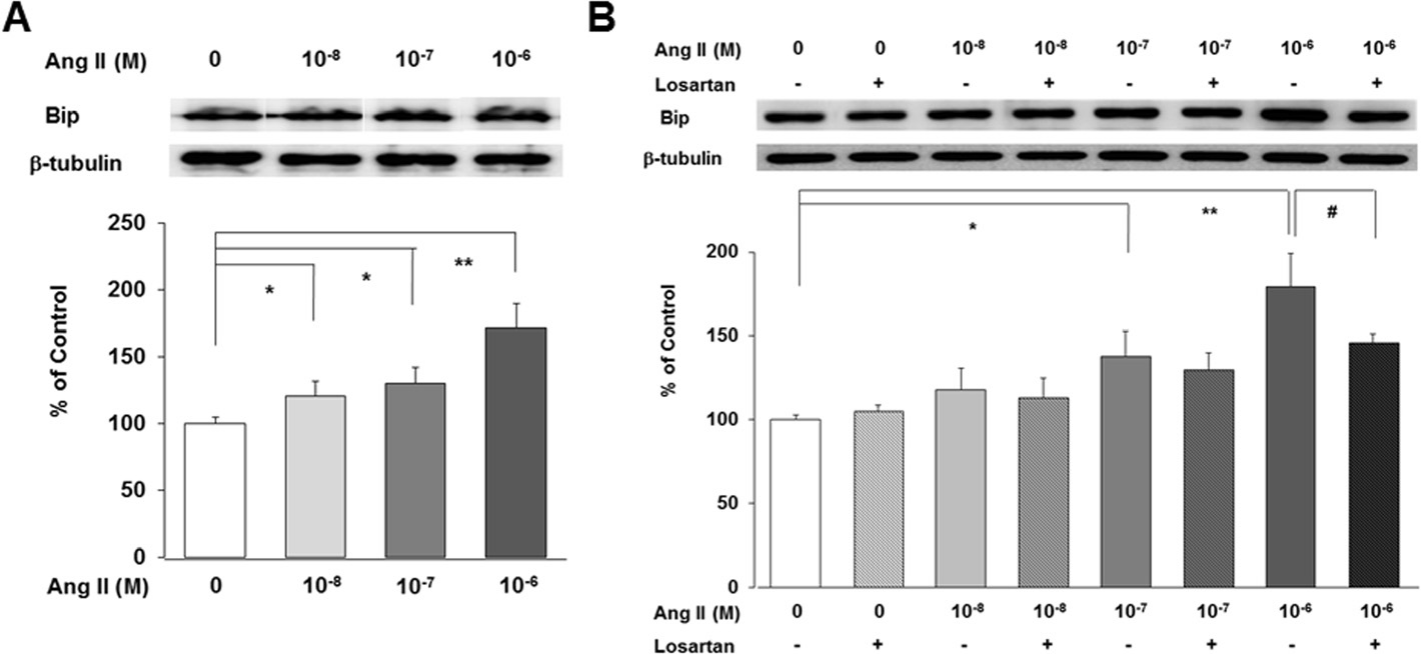
**Ang II induces ER stress in podocyte.** Ang II increases Bip protein, an ER chaperone, in a dose-dependent manner at 24 h **(A)**. However, losartan significantly ameliorates the upregulated Bip induced by higher does (10^−7^ M) of Ang II **(B)**. Data on the densitometric analysis of Bip/β-tubulin ratio are expressed as mean ± SD. Control (100%); the value of without Ang II. *P < 0.05 and **P < 0.01 versus control.

Ang II increased Bip protein, an ER chaperone, in a dose-dependent manner at 24 h after correcting for β-tubulin levels (*n* = 3, *P* < 0.05 and 0.01, Figure 1A). To assess the role of AT1R in the regulation of ER stress, we treated cells with 10^−6^ M losartan. Losartan significantly ameliorated the upregulated Bip induced by higher does (10^−7^ M) of Ang II after correcting for β-tubulin levels (*n* = 3, *P* < 0.05, Figure 1B).

**Figure 2:**
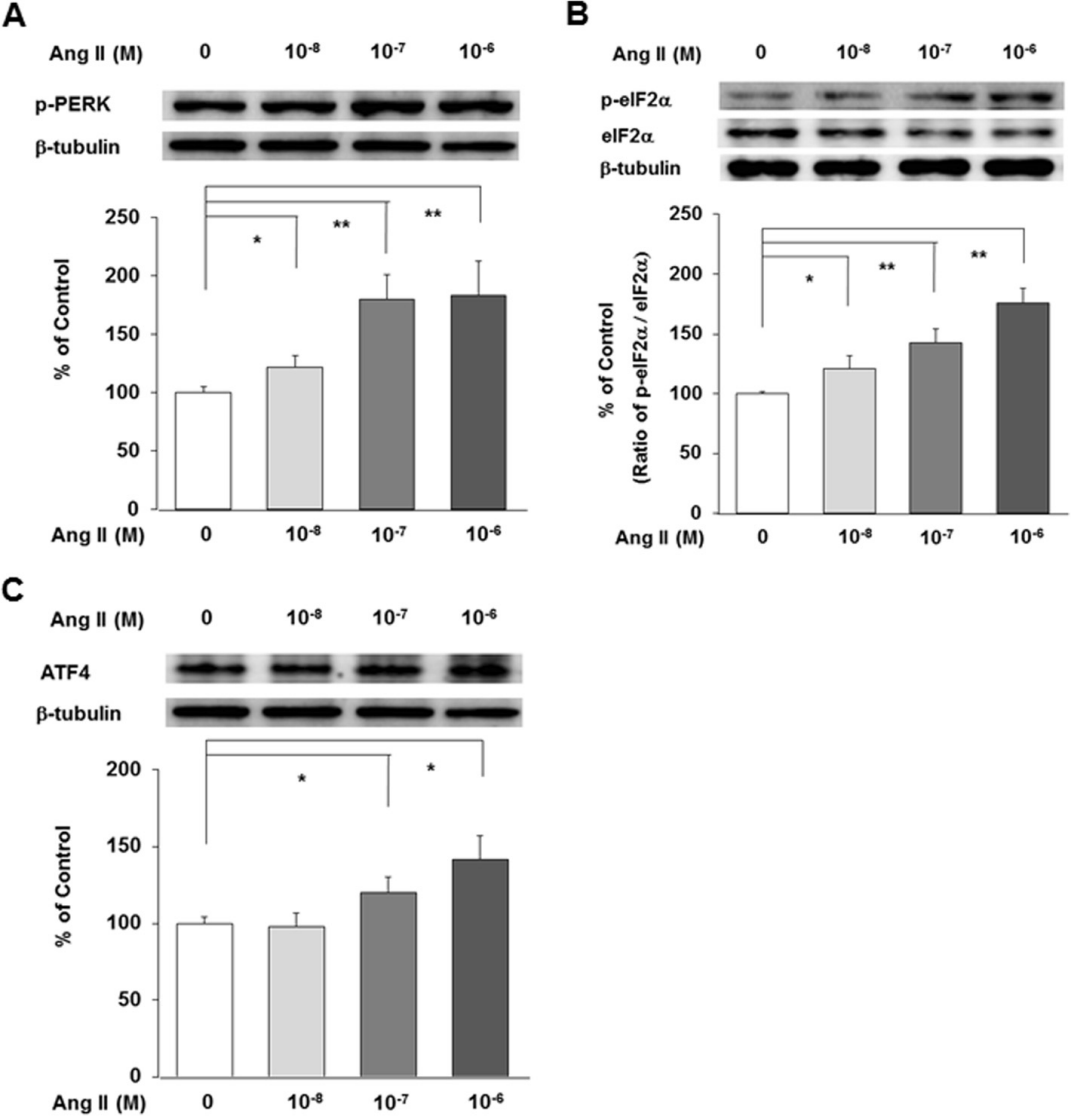
**Ang II increases ER stress proteins.** Ang II increased phospho-PERK **(A)**, phospho-eIF2α **(B)**, and ATF4 **(C)** significantly in a dose-dependent manner at 24 h. Ang II did not affect eIF2α but increased phospho-eIF2α **(B)**. Data on the densitometric analysis of phospho-PERK/β-tubulin ratio, phospho-eIF2α/total eIF2α ratio, and ATF4/β-tubulin ratio are expressed as mean ± SD, respectively. Control (100%); the value of without Ang II. *P < 0.05 and **P < 0.01 versus control.

Ang II upregulated ER stress proteins including phospho-PERK, phospho-eIF2α, and ATF4 proteins in a dose-dependent manner at 24 h after correcting for β-tubulin levels. Ang II increased phospho-PERK significantly in a dose-dependent manner at 24 h (*n* = 3, *P* < 0.05 and 0.01, Figure 2A). Ang II did not affect eIF2α but increased phospho-eIF2α, which resulted that Ang II upregulated phospho-eIF2α significantly in a dose-dependent manner after correcting for eIF2α or β-tubulin levels (*n* = 3, *P* < 0.05 and 0.01, Figure 2B). Ang II also increased ATF4 significantly at high doses at 24 h (*n* = 3, *P* < 0.05, Figure 2C).

### LY294002, a PI3-kinase inhibitor, augments Ang II-induced ER stress

**Figure 3:**
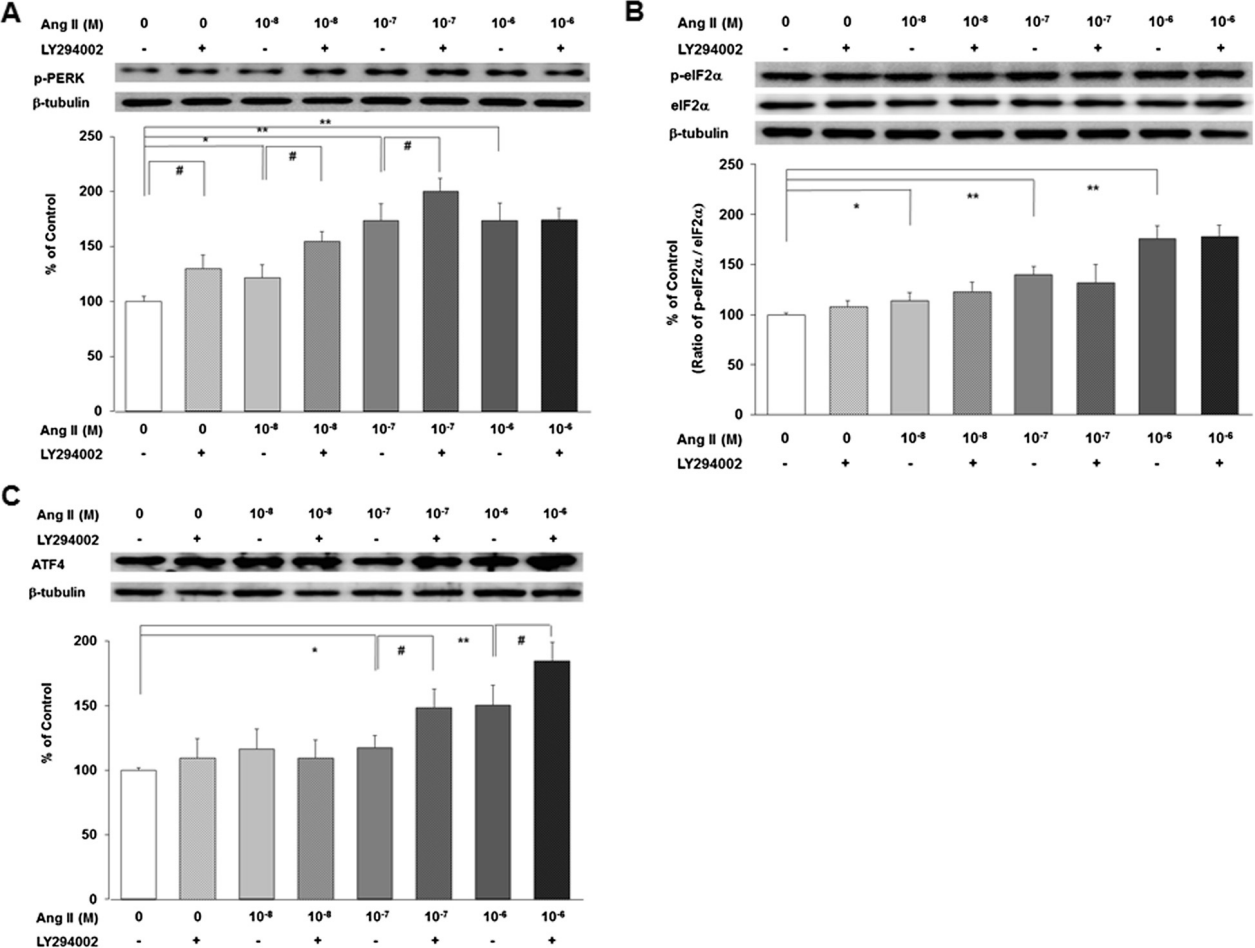
**LY294002, a PI3-kinase inhibitor, augments Ang II-induced ER stress.** LY294002 further augments the upregulated phospho-PERK induced by low doses of Ang II **(A)**. However, LY294002 does not affect phospho-eIF2α upregulated by Ang II **(B)**. LY294002 further magnified upregulation of ATF4 induced by high doses of Ang II **(C)**. Data on the densitometric analysis of each proteins/β-tubulin ratio are expressed as mean ± SD (*n* = 3). Control (100%); the value of no Ang II conditions. *P < 0.05 and **P < 0.01 versus control. ^#^ *P* < 0.05 versus the respective values without LY294002.

Similar to Figure 1, Ang II upregulated ER stress proteins, such as, phospho-PERK, phospho-eIF2α, and ATF4 proteins in a dose-dependent manner. LY294002 further augmented upregulation of phospho-PERK induced by low doses of Ang II (*n* = 3, *P* < 0.05, Figure 3A). However, LY294002 did not affect phospho-eIF2α upregulated by Ang II (*n* = 3, Figure 3B). LY294002 further magnified upregulation of ATF4 induced by high doses of Ang II (*n* = 3, *P* < 0.05, Figure 3C). Although the response to PI3-kinase inhibition is different to each ER stress proteins, PI3-kinase inhibition seems to augment the upregulated ER stress induced by Ang II.

## Discussion

Dysfunction of the UPR or prolonged ERS, disturbs ER homeostasis, leading to many human diseases, including neurodegenerative disease, metabolic disease, inflammatory disease, and diabetes mellitus [13]. It is important to elucidate the mechanisms by which UPR signaling contributes to pathogenesis of these diseases. Elucidation of the molecular mechanisms of ERS-related diseases may shed valuable light on these potential therapeutic targets. The induction of ER stress markers has been described in human kidney biopsies of different glomerulopathies, including membranous nephropathy, focal segmental glomerulosclerosis, and minimal change disease [16,17]. It has been reported that various stimuli can cause ER malfunction leading to ERS, such as ischemia, hypoxia, hyperglycemia, and heat shock [12-14,18]. In those stimuli, one common obvious thing is Ang II.

It has been reported that the Ang-II plays an unprecedented role in the pathogenesis of STZ-induced experimental diabetic nephropathy via the ERS-induced renal apoptosis [19-21]. As the involvement of ERS in the pathophysiology of diabetic nephropathy is relatively a new area of research, few studies are coming out. During the ERS, the tubular epithelial cells can undergo protective responses, but the persistent proteinuria and hyperglycemia can lead to an epithelial apoptosis [20]. Sun et al. [21] have reported that the ACE inhibitors considerably suppressed the ER stress-induced renal tubular apoptosis in STZ-induced experimental diabetic nephropathy model. Lakshmanan et al. [19] have also reported that the AT1R-specific blocker, olmesartan, blunted the upregulated GRP78 and ER-associated apoptosis proteins, such as TRAF2, IRE-1α, CHOP, p-JNK, and procaspase-12, in STZ-induced diabetic mice, which suggested that Ang II inhibition could be a potential therapy in treating ERS-induced renal apoptosis via the modulation of AT-1R/CHOP-JNK-Caspase-12 pathway in STZ-induced diabetic nephropathy.

ERS also plays an important role in podocyte injury. In congenital nephrotic syndrome (NS) model caused by mutational defect in CD2AP, there were dilatation of the membrane of rough ER and increased number of cytoplasmic empty-appearing vacuoles, implying inadequate protein folding ERS [22]. In another congenital NS model caused by mutational defect in α-actinin-4, there was upregulation of ER chaperones and ERS proteins and increased podocyte apoptosis [23]. Mutation of the GBM component laminin subunit β2 also leads to experimental NS, which is caused by defective secretion of laminin from podocytes to the GBM and is accompanied by podocyte ERS [24]. Thus, congenital NS with mutation of the glomerular filtration components is associated with the accumulation of mutated and misfolded proteins in the ER and disruption of the glomerular filtration structure, leading to proteinuria [25]. These findings highlight the possibility that UPR augmentation therapy might be used to enhance ER proteostasis as a new therapeutic approach to congenital NS.

ERS is the common finding under various pathogenic microenvironments, contributing to the progression of various podocyte diseases. Abnormal protein accumulation associated with ERS in the ER of podocytes produces structural and functional damage in the cells, which in turn leads to severe proteinuria. Podocytes of transgenic rats overexpressing a mutant megsin, without the capacity for polymerization within the ER, do not exhibit ERS or podocyte damage, suggesting a pathogenic role of ER retention of polymerized megsin [26]. In another albumin overload studies, a mimic of proteinuria, calcium entry via transient receptor protein 6 (TRPC6) or downregulation of CD2AP by albumin overload induced UPR-mediated apoptosis in podocytes [22,27].

In an experimental diabetic nephropathy model, three hallmarks of ER-associated apoptosis, C/EBP homologous protein (CHOP), c-Jun NH_2_-terminal kinase (JNK), and caspase-12, were found to have activated, suggesting that apoptosis induced by ERS occurred in diabetic kidney, which may contribute to the development of diabetic nephropathy [28]. ERS also contributes to podocyte injury caused by increased expression of monocyte chemoattractant protein 1 (MCP-1), which has a central role in the inflammation associated with diabetic nephropathy [29]. In experimental diabetic nephropathy and NS models, activation of rapamycin-sensitive protein kinase complex TORC1, which contributes to multiple cellular processes associated with proteostasis, also triggers UPR activation in podocytes, leading to podocyte injury [30,31]. Taken together, these findings demonstrate that not only are mutations in podocyte protein folding associated with ERS but acquired renal diseases that induce podocyte injury are as well. Our data also demonstrated that Ang II, a major podocyte injury inducer, could induce podocyte ERS of PERK-eIF2α-ATF4 axis.

In podocyte, CD2AP and nephrin interact with a subunit of PI3-kinase, and subsequently stimulate the PI3-kinase-dependent activation of the intracellular Akt kinase pathway, which is necessary for the regulation of actin dynamics and the cell survival [32,33]. In human cancer cells, inhibition of lipid raft-associated PI3-kinase/PKB (Akt) signaling by TSWU-CD4, a synthetic bichalcone analog, induced ERS- and oligomeric Bax/Bak-mediated apoptosis, which were substantially reversed by overexpression of the wt PI3-kinase p85α subunit. Therefore, the suppression of lipid raft-associated PI3-kinase/Akt signaling could induce the ERS and subsequent apoptosis [34]. In mouse pancreatic beta cells (NIT-1 cells), metformin directly protects against dysfunction and death of ERS via AMP-activated protein kinase (AMPK) and PI-3 kinase activation, which were abolished by AMPK and PI3-kinase inhibitors [35].

Interestingly, Mounir Z, et al. [36] reported that either pharmacological inhibition of the PI3-kinase/Akt pathway or genetic or small interfering RNA-mediated ablation of Akt could induce the phosphorylation of eIF2a and PERK in mammalian cells and Drosophila cells. The activity of PERK and the abundance of phospho-eIF2α were reduced in mouse mammary gland tumors that contained activated Akt, as well as in cells exposed to ERS or oxidative stress; however, in unstressed cells, the PERK-eIF2α pathway mediated survival and facilitated adaptation to the deleterious effects of the inactivation of PI3-K or Akt. Inactivation of the PERK-eIF2α pathway increased the susceptibility of tumor cells to death by pharmacological inhibitors of PI3-K or Akt. Thus, they suggested that the PERK-eIF2α pathway would provide an important link between Akt signaling and translational control, which had implications for tumor formation and treatment. Taken together, inhibition of PI3-kinase/Akt signaling could induce the ERS and subsequent cell death.

## Conclusion

**Figure 4:**
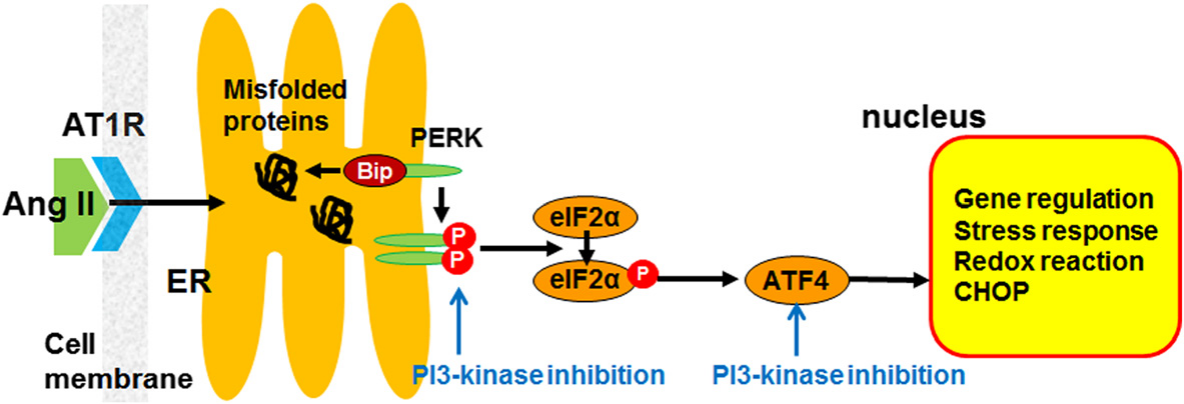
**Schematic view of ER stress pathway induced by Ang II in podocyte.** Ang II could induce podocyte ER stress of PERK-eIF2α-ATF4 axis via PI3-kinase pathway. Arrows mean induction or augmentation. Ang II, angiotensin II; AT1R, angiotensin II type 1 receptor; ATF6, activating transcription factor 6; Bip, binding immunoglobulin protein; eIF2α, eukaryotic translation initiation factor 2cme; ER, endoplasmic reticulum; PERK, PKR-like ER kinase.

In this study, PI3-kinase inhibition augmented Ang II-induced podocyte ERS via PERK-eIF2α pathway, which may be related to Ang II-induced podocyte damage (Figure 4). Further study on the pathogenic relationship between ERS and apoptosis in Ang II-induced podocyte injury would be needed. In conclusion, Ang II could induce podocyte ERS via PI3-kinase pathway, which would contribute to the development of podocyte injury induced by Ang II, and the augmentation of PI3-kinase would be a novel therapeutic target of Ang II-induced podocyte dysfunction.
